# Manic-Depressive Psychosis

**Published:** 1918-10

**Authors:** T. L. Moody

**Affiliations:** San Antonio, Texas


### Manic-Depressive Psychosis.

T. L. MOODY, M. D., SAN ANTONIO, TEXAS.

In discussing this subject it is not my intention to try to go into any new theory, but to discuss it from a practical standpoint, based upon clinical observations made within the last thirteen years, during which time I have been interested in this class of work, having in mind all through the discussion, a certain class of cases which we seldom ever hear discussed entirely in a class to themselves.

In about 1650 cases of Psychoses taken from our case records I have selected 142 cases which we have diagnosed Manic-Depressive Psychosis. In selecting these cases I have not included Involution Psychosis, Puerpal Insanities and other toxic cases, such as Pellagra, Post-febrile cases, etc. The cases selected are between the ages of 15 and 37 years, not a single case over 36 years of age am I reporting. The reason I have for doing this is to be sure that I do not infringe in the least on the cases which could be diagnosed differently, and also to bring out the fact that we should have a better understanding as to the cause of this diseased condition.

There is no question but that heredity plays quite an important part in this disease, and with Dementia Praecox, is classed as presenting its heredity in the collateral branches of the family more often than any of the other forms of mental disease. So in the statistics given later we must bear this in mind.

From these 142 cases we were able to get a clear history of heredity in 39 per cent. of them, and in about 59 per cent. we were given a negative hereditary history as to mental disease proper, and in about 2 per cent. we were unable to get history concerning heredity. This seems to show that heredity is not so great in this class of cases as might be supposed, but we must take into consideration the fact that relatives of patients will not give history as they should and in fact will deny any heredity at all in a great many instances; especially, are we unable to get history when it occurs in distant relatives as it does in this disease. In figuring the ratio of male to female, I find in our cases 32 per cent. male and about 68 per cent. female, and of these married females 37 per cent., single females 30 per cent.; married males 9 per cent., single males 24 per cent.

I have gathered from a few annual reports of State Institutions for Insane a number of cases of Manic-Depressive Insanity and find that 55 per cent. of cases are female and 45 per cent. are male. These statistics differ from ours but can be explained I think, by the fact that all cases of ours are pay patients; therefore, the husband, in many instances, remains at home and by so doing is able to send his wife for treatment, while in the same family should the husband be the victim of disease, he would remain at home until it became absolutely necessary to be restrained, and probably go to the State Institution, or commit suicide before hand.

The same explanation will hold good to a certain extent in regard to married or single men, as you can see quite a difference in percentage of cases treated. We will also keep in mind that many have diagnosed their Involution cases and toxic insanities as Manic-Depressive Insanity.

Syphilis, both acquired and hereditary, is given as a cause for this disease, but I do not believe myself that we will find as much of it in this class of cases as in many other of the Psychoses. I also believe there are cases of Manic-Depressive Insanity whose blood will show a positive Wasserman reaction and still Syphilis has absolutely no bearing on the case. We have treated cases before the time of Wasserman reaction, which I am practically sure had Syphilis to the extent of giving a positive reaction, had one been done. These cases recovered without any anti-Syphilitic treatment whatever. We had given others Mercury with little or no apparent effect. I am one who does not believe every one has Syphilis, while I fully realize the importance of looking for it. I feel that we should not depend solely upon anti-Syphilitic treatment to do the work, then if it does not do it, be content to give up and send the patient to the Asylum, for that is usually what we do when we decide the patient is incurable. I am glad to say in this connection, however, that the State Institutions are doing a much more scientific work all along in these cases, and will be able to do more from time to time, as the Public in general is realizing that Insanity is a diseased condition and should be treated as such. We have been thinking for a few years that the human race had undergone marked degeneration, but as stated by Dr. Lewellys F. Barker, of Baltimore, in an article written for the Journal of Nervous and Mental Disease, this theory is quite discredited from the fact that the men in the war and out of it in the belligerent countries are standing the strain of same better than ever before, which shows that the human nervous system is better able today to stand strain than ever before in its history. This fact is probably due to a better way of living and understanding, gained through advice from people who are in position to know best, which has made stronger nervous systems generally. I can hardly believe that we as a whole are degenerating, and am not willing to take that view of it as yet.

We meet with cases where we can seemingly trace the cause entirely to some strain, either physical or mental, or to some sudden shock, but if gone into thoroughly this will prove to be only the exciting cause precipitating the attack. In a patient with a more stable nervous organization the attack would not have developed, which shows to us conclusively that heredity is most certainly a very important factor. While, as stated above, it may not be, and most likely is not, directly hereditary. The patient inherits a weakened nervous makeup, which will handicap him considerably, without help and advice from someone who knows more about these conditions than he does, and this is where the family physician can be of material benefit to his patients, having studied their temperaments and environments. This advice should be given to the relatives of patients and should be carried out thoroughly in order to get the best results. I am sure that I have seen cases develop which could have been prevented. I have in mind a young man who was tried for murder just a short time ago, and who came clear all right, but the case had been postponed from time to time for three years, until the man gave way under the strain of it all, and just after the trial he had a very violent outbreak of Manic-Depressive Insanity; while, if he could have had his trial in a reasonable length of time he would never have had the attack.

The symptoms presented in this disease are very numerous, from the fact that they arise from so many different sources. I am not going into all of them as the text books do, and will only mention a few of the general symptoms. The main symptoms are the depression and maniacal excitement or exaltation. With depression, of course, we see the melancholy state with despondency and pessimism in a very severe form. Excited cases and maniacally turned are very noisy and confused in some instances, and in others are very elated. We also see cases who present the mixed type of symptoms. Also we must be able to recognize constitutional symptoms in these cases in order to make an intelligent diagnosis. I mean by this the personal equation of the patient must be reckoned with as much as the disease itself. Some individuals present symptoms of maniacal nature, such as singing and laughing; while others will be disturbed from fear and become quite emotional, and still have the same diseased condition.

It is very important to differentiate this from other conditions, such as General Paresis and Dementia Praecox. This sometimes is difficult to do in the beginning as the symptoms are quite similar. We have a much smaller per cent. of recoveries in Dementia Praecox, and all cases of Paresis are said to be incurable; therefore, it means much to the patient's relatives if we can give them a trustworthy opinion in making our diagnosis. One who is accustomed to seeing these cases can usually be quite positive, however, as there are symptoms in common in each disease which will determine the condition. In Dementia Praecox we have the Psychical infeeblement, failure of interest, judgment affected, emotional deterioration, negativism, and sterotypy. In Paresis we have the pupilary disturbances, and we usually get a positive Wasserman and a certain amount of mental weakness, which will give us an insight into the disease. In manic depressive cases we have the exalted mood, acclerated flow of ideas, motor excitement in the manic type, and in the depressed type retarded flow of thought, motor inhibition, and suicidal tendencies.

I find in our cases that about 30 per cent. were maniacal, about 47 per cent. depressed, and 23 per cent. were the mixed type. So we can see from this what various symptoms we may encounter in this disease.

The depressed cases of this disease are very suicidal and are more liable to carry out their intentions than those suffering from depression in the other psychoses, such as Involutional Melancholia and Pellagra. Therefore, they should be kept constantly under the care of a competent nurse.

We also meet with recurrences frequently, some authorities believe all cases recur sometime during life, but I do not believe this, if the case can have the proper advice and management. The percentage of previous attacks in our cases I find is about 44 and the average age of those who had had previous attacks was 28½ years. I believe those who are able to secure the best care and treatment early enough and continue it long enough, the prognosis is better than most of us are willing to admit. We find in the treatment of cases we have a great deal of trouble in convincing the patient, also relatives of patients, that it is necessary to remain under treatment sufficiently long to get the best results. As soon as the very acute symptoms are relieved they feel that the patient is well, which, of course, is a very erronous idea, and the following statistics will bear me out in this. In this series of 142 cases referred to above, 30 were discharged unimproved, and the average length of time these 30 cases were under treatment was about one month, and none for more than four months. Of the other 112 cases who recover or improved, the average length of time under treatment was a little more than three months. If in all of these cases the average length of time under treatment could have been somewhat increased, I feel sure that the percentage of improvements in the unimproved would have been increased quite a good deal, and that the number of recoveries in the improved and recovered group would have been greater.

In the way of medication, I use nerve tonics and drugs to favor elimination. Wet packs and baths have been very satisfactory in my hands as a means of quieting and producing sleep in these cases, and I use just as little drugs as possible for this purpose. In the way of drugs for hypnotics, I think trional is one of the very best. I consider all medication of little value, unless we have first the conditions of rest and isolation.

